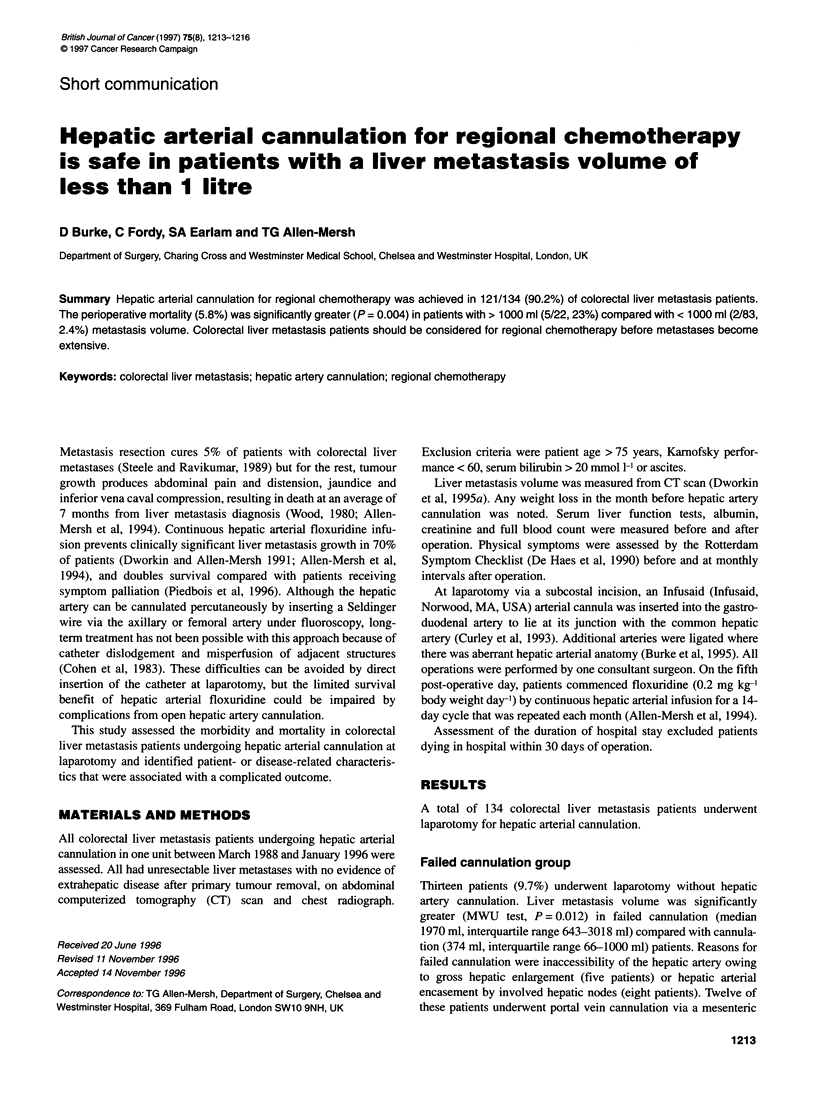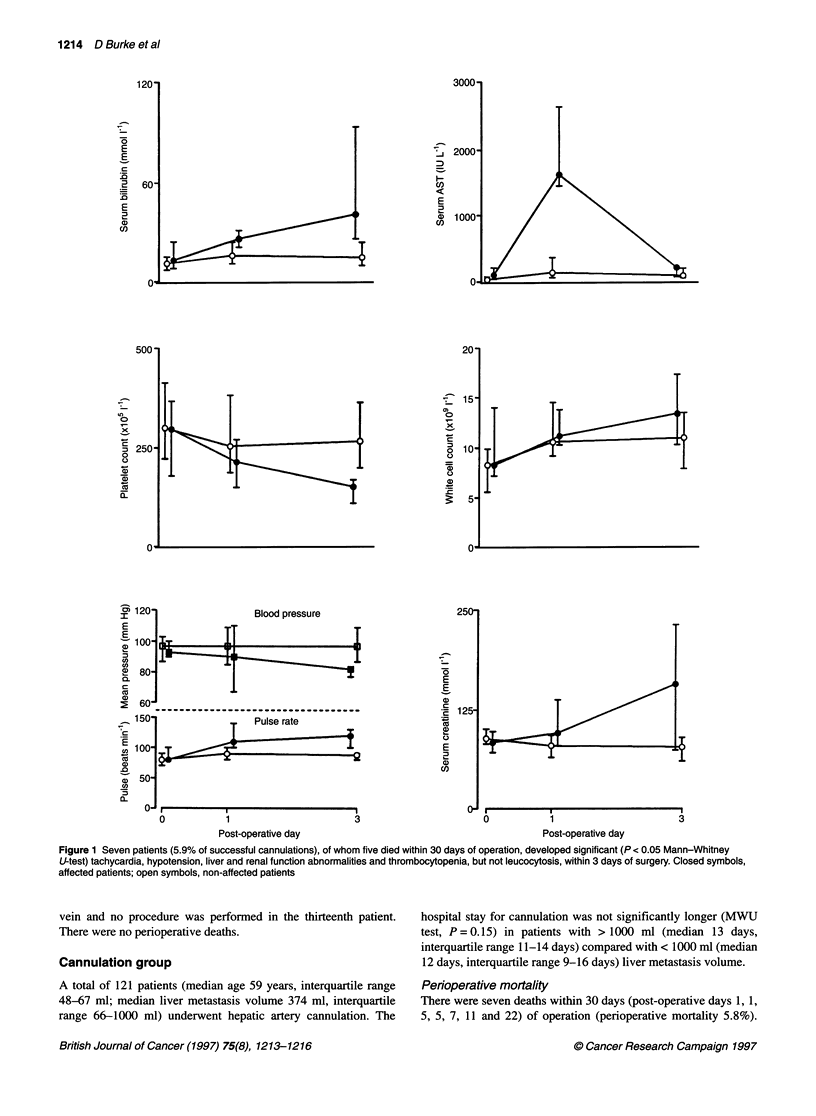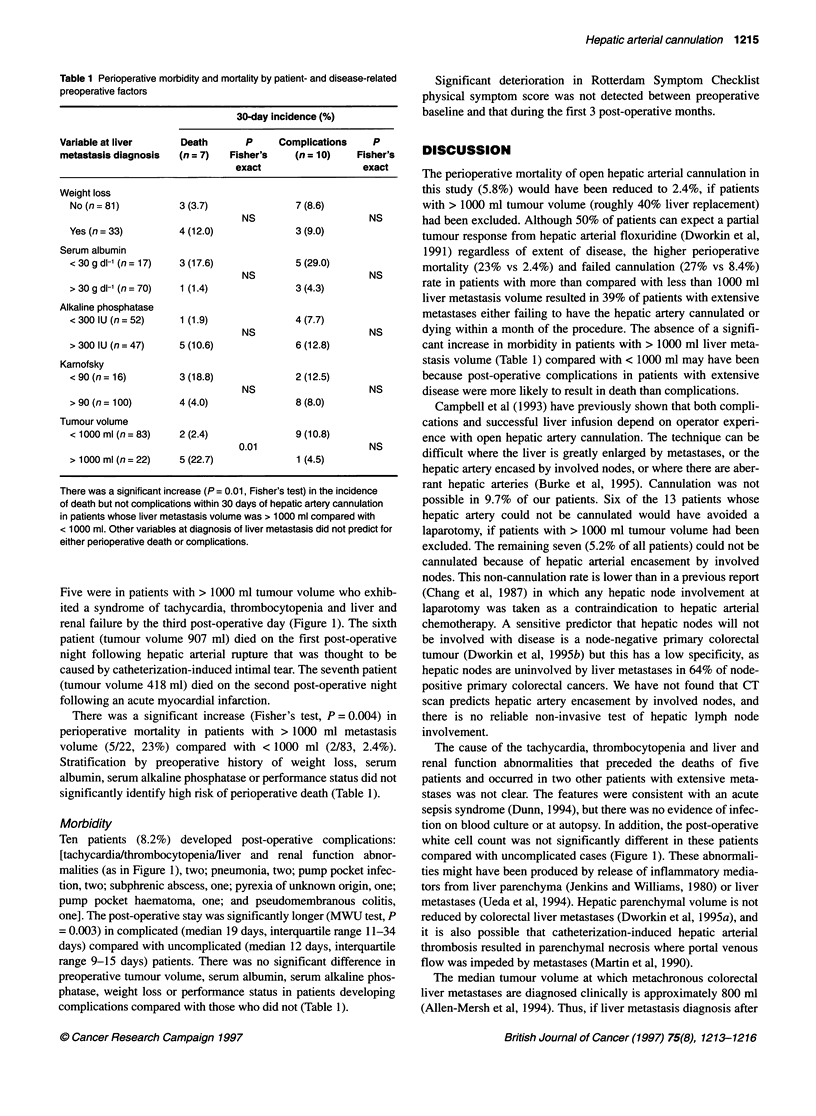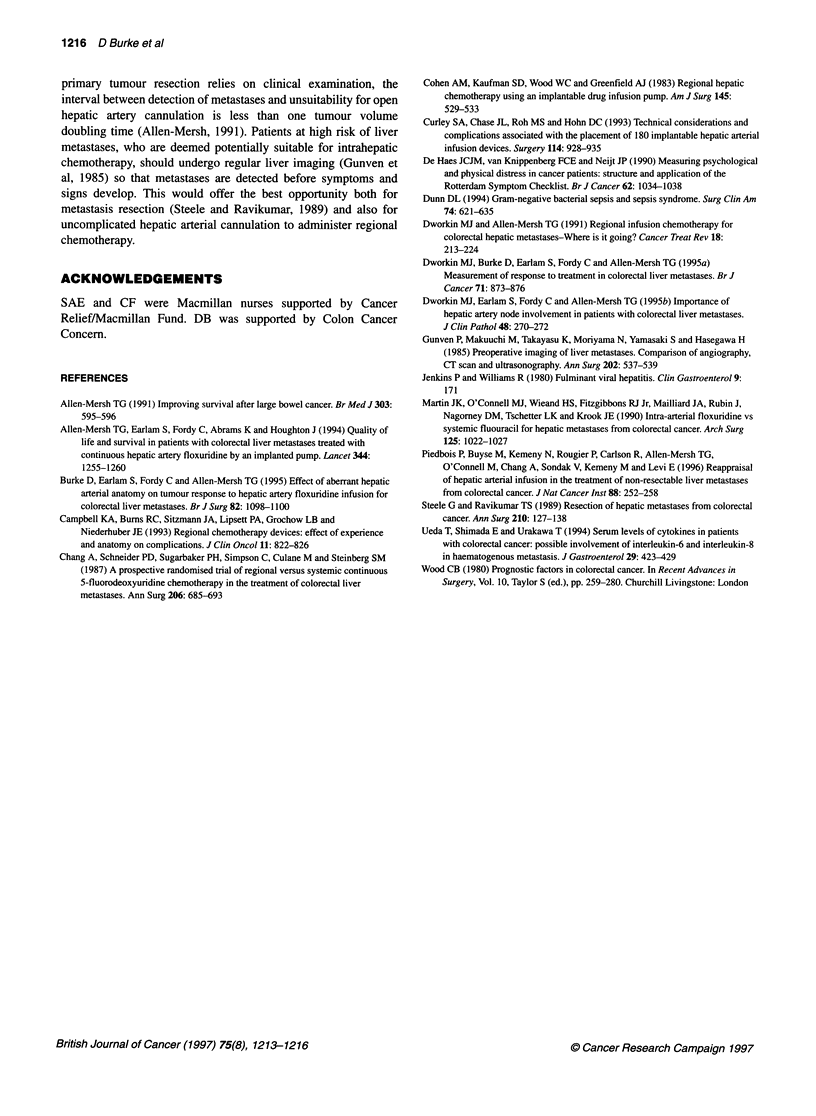# Hepatic arterial cannulation for regional chemotherapy is safe in patients with a liver metastasis volume of less than 1 litre.

**DOI:** 10.1038/bjc.1997.207

**Published:** 1997

**Authors:** D. Burke, C. Fordy, S. A. Earlam, T. G. Allen-Mersh

**Affiliations:** Department of Surgery, Charing Cross and Westminster Medical School, Chelsea and Westminster Hospital, London, UK.

## Abstract

Hepatic arterial cannulation for regional chemotherapy was achieved in 121/134 (90.2%) of colorectal liver metastasis patients. The perioperative mortality (5.8%) was significantly greater (P = 0.004) in patients with > 1000 ml (5/22, 23%) compared with < 1000 ml (2/83, 2.4%) metastasis volume. Colorectal liver metastasis patients should be considered for regional chemotherapy before metastases become extensive.


British Journal of Cancer (1997) 75(8), 1213–1216
© 1997 Cancer Research Campaign

Short communication

### Hepatic arterial cannulation for regional chemotherapy is safe in patients with a liver metastasis volume of less than 1 litre

**D Burke, C Fordy, SA Earlam and TG Allen-Mersh**

Department of Surgery, Charing Cross and Westminster Medical School, Chelsea and Westminster Hospital, London, UK

**Summary** Hepatic arterial cannulation for regional chemotherapy was achieved in 121/134 (90.2%) of colorectal liver metastasis patients. The perioperative mortality (5.8%) was significantly greater ($P = 0.004$) in patients with > 1000 ml (5/22, 23%) compared with < 1000 ml (2/83, 2.4%) metastasis volume. Colorectal liver metastasis patients should be considered for regional chemotherapy before metastases become extensive.

**Keywords:** colorectal liver metastasis; hepatic artery cannulation; regional chemotherapy

Metastasis resection cures 5% of patients with colorectal liver metastases (Steele and Ravikumar, 1989) but for the rest, tumour growth produces abdominal pain and distension, jaundice and inferior vena caval compression, resulting in death at an average of 7 months from liver metastasis diagnosis (Wood, 1980; Allen-Mersh et al, 1994). Continuous hepatic arterial floxuridine infusion prevents clinically significant liver metastasis growth in 70% of patients (Dworkin and Allen-Mersh 1991; Allen-Mersh et al, 1994), and doubles survival compared with patients receiving symptom palliation (Piedbois et al, 1996). Although the hepatic artery can be cannulated percutaneously by inserting a Seldinger wire via the axillary or femoral artery under fluoroscopy, long-term treatment has not been possible with this approach because of catheter dislodgement and misperfusion of adjacent structures (Cohen et al, 1983). These difficulties can be avoided by direct insertion of the catheter at laparotomy, but the limited survival benefit of hepatic arterial floxuridine could be impaired by complications from open hepatic artery cannulation.

This study assessed the morbidity and mortality in colorectal liver metastasis patients undergoing hepatic arterial cannulation at laparotomy and identified patient- or disease-related characteristics that were associated with a complicated outcome.

### MATERIALS AND METHODS

All colorectal liver metastasis patients undergoing hepatic arterial cannulation in one unit between March 1988 and January 1996 were assessed. All had unresectable liver metastases with no evidence of extrahepatic disease after primary tumour removal, on abdominal computerized tomography (CT) scan and chest radiograph. Exclusion criteria were patient age > 75 years, Karnofsky performance < 60, serum bilirubin > 20 mmol $l^{-1}$ or ascites.

Liver metastasis volume was measured from CT scan (Dworkin et al, 1995*a*). Any weight loss in the month before hepatic artery cannulation was noted. Serum liver function tests, albumin, creatinine and full blood count were measured before and after operation. Physical symptoms were assessed by the Rotterdam Symptom Checklist (De Haes et al, 1990) before and at monthly intervals after operation.

At laparotomy via a subcostal incision, an Infusaid (Infusaid, Norwood, MA, USA) arterial cannula was inserted into the gastroduodenal artery to lie at its junction with the common hepatic artery (Curley et al, 1993). Additional arteries were ligated where there was aberrant hepatic arterial anatomy (Burke et al, 1995). All operations were performed by one consultant surgeon. On the fifth post-operative day, patients commenced floxuridine (0.2 mg $kg^{-1}$ body weight $day^{-1}$) by continuous hepatic arterial infusion for a 14-day cycle that was repeated each month (Allen-Mersh et al, 1994).

Assessment of the duration of hospital stay excluded patients dying in hospital within 30 days of operation.

### RESULTS

A total of 134 colorectal liver metastasis patients underwent laparotomy for hepatic arterial cannulation.

### Failed cannulation group

Thirteen patients (9.7%) underwent laparotomy without hepatic artery cannulation. Liver metastasis volume was significantly greater (MWU test, $P = 0.012$) in failed cannulation (median 1970 ml, interquartile range 643–3018 ml) compared with cannulation (374 ml, interquartile range 66–1000 ml) patients. Reasons for failed cannulation were inaccessibility of the hepatic artery owing to gross hepatic enlargement (five patients) or hepatic arterial encasement by involved hepatic nodes (eight patients). Twelve of these patients underwent portal vein cannulation via a mesenteric

*Received 20 June 1996*
*Revised 11 November 1996*
*Accepted 14 November 1996*

*Correspondence to:* TG Allen-Mersh, Department of Surgery, Chelsea and Westminster Hospital, 369 Fulham Road, London SW10 9NH, UK

**Figure 1** Seven patients (5.9% of successful cannulations), of whom five died within 30 days of operation, developed significant ($P < 0.05$ Mann–Whitney *U*-test) tachycardia, hypotension, liver and renal function abnormalities and thrombocytopenia, but not leucocytosis, within 3 days of surgery. Closed symbols, affected patients; open symbols, non-affected patients vein and no procedure was performed in the thirteenth patient. There were no perioperative deaths.

### Cannulation group

A total of 121 patients (median age 59 years, interquartile range 48–67 ml; median liver metastasis volume 374 ml, interquartile range 66–1000 ml) underwent hepatic artery cannulation. The hospital stay for cannulation was not significantly longer (MWU test, $P = 0.15$) in patients with > 1000 ml (median 13 days, interquartile range 11–14 days) compared with < 1000 ml (median 12 days, interquartile range 9–16 days) liver metastasis volume.

### *Perioperative mortality*

There were seven deaths within 30 days (post-operative days 1, 1, 5, 5, 7, 11 and 22) of operation (perioperative mortality 5.8%).

© Cancer Research Campaign 1997

**Table 1** Perioperative morbidity and mortality by patient- and disease-related preoperative factors

| Variable at liver metastasis diagnosis | 30-day incidence (%) Death ($n$ = 7) | $P$ Fisher's exact | Complications ($n$ = 10) | $P$ Fisher's exact |
|---|---|---|---|---|
| Weight loss | | | | |
| No ($n$ = 81) | 3 (3.7) | NS | 7 (8.6) | NS |
| Yes ($n$ = 33) | 4 (12.0) | | 3 (9.0) | |
| Serum albumin | | | | |
| < 30 g dl$^{-1}$ ($n$ = 17) | 3 (17.6) | NS | 5 (29.0) | NS |
| > 30 g dl$^{-1}$ ($n$ = 70) | 1 (1.4) | | 3 (4.3) | |
| Alkaline phosphatase | | | | |
| < 300 IU ($n$ = 52) | 1 (1.9) | NS | 4 (7.7) | NS |
| > 300 IU ($n$ = 47) | 5 (10.6) | | 6 (12.8) | |
| Karnofsky | | | | |
| < 90 ($n$ = 16) | 3 (18.8) | NS | 2 (12.5) | NS |
| > 90 ($n$ = 100) | 4 (4.0) | | 8 (8.0) | |
| Tumour volume | | | | |
| < 1000 ml ($n$ = 83) | 2 (2.4) | 0.01 | 9 (10.8) | NS |
| > 1000 ml ($n$ = 22) | 5 (22.7) | | 1 (4.5) | |

There was a significant increase ($P$ = 0.01, Fisher's test) in the incidence of death but not complications within 30 days of hepatic artery cannulation in patients whose liver metastasis volume was > 1000 ml compared with < 1000 ml. Other variables at diagnosis of liver metastasis did not predict for either perioperative death or complications.

Five were in patients with > 1000 ml tumour volume who exhibited a syndrome of tachycardia, thrombocytopenia and liver and renal failure by the third post-operative day (Figure 1). The sixth patient (tumour volume 907 ml) died on the first post-operative night following hepatic arterial rupture that was thought to be caused by catheterization-induced intimal tear. The seventh patient (tumour volume 418 ml) died on the second post-operative night following an acute myocardial infarction.

There was a significant increase (Fisher's test, $P$ = 0.004) in perioperative mortality in patients with > 1000 ml metastasis volume (5/22, 23%) compared with < 1000 ml (2/83, 2.4%). Stratification by preoperative history of weight loss, serum albumin, serum alkaline phosphatase or performance status did not significantly identify high risk of perioperative death (Table 1).

### *Morbidity*

Ten patients (8.2%) developed post-operative complications: [tachycardia/thrombocytopenia/liver and renal function abnormalities (as in Figure 1), two; pneumonia, two; pump pocket infection, two; subphrenic abscess, one; pyrexia of unknown origin, one; pump pocket haematoma, one; and pseudomembranous colitis, one]. The post-operative stay was significantly longer (MWU test, $P$ = 0.003) in complicated (median 19 days, interquartile range 11–34 days) compared with uncomplicated (median 12 days, interquartile range 9–15 days) patients. There was no significant difference in preoperative tumour volume, serum albumin, serum alkaline phosphatase, weight loss or performance status in patients developing complications compared with those who did not (Table 1).

Significant deterioration in Rotterdam Symptom Checklist physical symptom score was not detected between preoperative baseline and that during the first 3 post-operative months.

### DISCUSSION

The perioperative mortality of open hepatic arterial cannulation in this study (5.8%) would have been reduced to 2.4%, if patients with > 1000 ml tumour volume (roughly 40% liver replacement) had been excluded. Although 50% of patients can expect a partial tumour response from hepatic arterial floxuridine (Dworkin et al, 1991) regardless of extent of disease, the higher perioperative mortality (23% vs 2.4%) and failed cannulation (27% vs 8.4%) rate in patients with more than compared with less than 1000 ml liver metastasis volume resulted in 39% of patients with extensive metastases either failing to have the hepatic artery cannulated or dying within a month of the procedure. The absence of a significant increase in morbidity in patients with > 1000 ml liver metastasis volume (Table 1) compared with < 1000 ml may have been because post-operative complications in patients with extensive disease were more likely to result in death than complications.

Campbell et al (1993) have previously shown that both complications and successful liver infusion depend on operator experience with open hepatic artery cannulation. The technique can be difficult where the liver is greatly enlarged by metastases, or the hepatic artery encased by involved nodes, or where there are aberrant hepatic arteries (Burke et al, 1995). Cannulation was not possible in 9.7% of our patients. Six of the 13 patients whose hepatic artery could not be cannulated would have avoided a laparotomy, if patients with > 1000 ml tumour volume had been excluded. The remaining seven (5.2% of all patients) could not be cannulated because of hepatic arterial encasement by involved nodes. This non-cannulation rate is lower than in a previous report (Chang et al, 1987) in which any hepatic node involvement at laparotomy was taken as a contraindication to hepatic arterial chemotherapy. A sensitive predictor that hepatic nodes will not be involved with disease is a node-negative primary colorectal tumour (Dworkin et al, 1995*b*) but this has a low specificity, as hepatic nodes are uninvolved by liver metastases in 64% of node-positive primary colorectal cancers. We have not found that CT scan predicts hepatic artery encasement by involved nodes, and there is no reliable non-invasive test of hepatic lymph node involvement.

The cause of the tachycardia, thrombocytopenia and liver and renal function abnormalities that preceded the deaths of five patients and occurred in two other patients with extensive metastases was not clear. The features were consistent with an acute sepsis syndrome (Dunn, 1994), but there was no evidence of infection on blood culture or at autopsy. In addition, the post-operative white cell count was not significantly different in these patients compared with uncomplicated cases (Figure 1). These abnormalities might have been produced by release of inflammatory mediators from liver parenchyma (Jenkins and Williams, 1980) or liver metastases (Ueda et al, 1994). Hepatic parenchymal volume is not reduced by colorectal liver metastases (Dworkin et al, 1995*a*), and it is also possible that catheterization-induced hepatic arterial thrombosis resulted in parenchymal necrosis where portal venous flow was impeded by metastases (Martin et al, 1990).

The median tumour volume at which metachronous colorectal liver metastases are diagnosed clinically is approximately 800 ml (Allen-Mersh et al, 1994). Thus, if liver metastasis diagnosis after

© Cancer Research Campaign 1997

primary tumour resection relies on clinical examination, the interval between detection of metastases and unsuitability for open hepatic artery cannulation is less than one tumour volume doubling time (Allen-Mersh, 1991). Patients at high risk of liver metastases, who are deemed potentially suitable for intrahepatic chemotherapy, should undergo regular liver imaging (Gunven et al, 1985) so that metastases are detected before symptoms and signs develop. This would offer the best opportunity both for metastasis resection (Steele and Ravikumar, 1989) and also for uncomplicated hepatic arterial cannulation to administer regional chemotherapy.

### ACKNOWLEDGEMENTS

SAE and CF were Macmillan nurses supported by Cancer Relief/Macmillan Fund. DB was supported by Colon Cancer Concern.

### REFERENCES

Allen-Mersh TG (1991) Improving survival after large bowel cancer. *Br Med J* **303**: 595–596

Allen-Mersh TG, Earlam S, Fordy C, Abrams K and Houghton J (1994) Quality of life and survival in patients with colorectal liver metastases treated with continuous hepatic artery floxuridine by an implanted pump. *Lancet* **344**: 1255–1260

Burke D, Earlam S, Fordy C and Allen-Mersh TG (1995) Effect of aberrant hepatic arterial anatomy on tumour response to hepatic artery floxuridine infusion for colorectal liver metastases. *Br J Surg* **82**: 1098–1100

Campbell KA, Burns RC, Sitzmann JA, Lipsett PA, Grochow LB and Niederhuber JE (1993) Regional chemotherapy devices: effect of experience and anatomy on complications. *J Clin Oncol* **11**: 822–826

Chang A, Schneider PD, Sugarbaker PH, Simpson C, Culane M and Steinberg SM (1987) A prospective randomised trial of regional versus systemic continuous 5-fluorodeoxyuridine chemotherapy in the treatment of colorectal liver metastases. Ann Surg **206**: 685–693

Cohen AM, Kaufman SD, Wood WC and Greenfield AJ (1983) Regional hepatic chemotherapy using an implantable drug infusion pump. *Am J Surg* **145**: 529–533

Curley SA, Chase JL, Roh MS and Hohn DC (1993) Technical considerations and complications associated with the placement of 180 implantable hepatic arterial infusion devices. *Surgery* **114**: 928–935

De Haes JCJM, van Knippenberg FCE and Neijt JP (1990) Measuring psychological and physical distress in cancer patients: structure and application of the Rotterdam Symptom Checklist. *Br J Cancer* **62**: 1034–1038

Dunn DL (1994) Gram-negative bacterial sepsis and sepsis syndrome. *Surg Clin Am* **74**: 621–635

Dworkin MJ and Allen-Mersh TG (1991) Regional infusion chemotherapy for colorectal hepatic metastases–Where is it going? *Cancer Treat Rev* **18**: 213–224

Dworkin MJ, Burke D, Earlam S, Fordy C and Allen-Mersh TG (1995*a*) Measurement of response to treatment in colorectal liver metastases. *Br J Cancer* **71**: 873–876

Dworkin MJ, Earlam S, Fordy C and Allen-Mersh TG (1995*b*) Importance of hepatic artery node involvement in patients with colorectal liver metastases. *J Clin Pathol* **48**: 270–272

Gunven P, Makuuchi M, Takayasu K, Moriyama N, Yamasaki S and Hasegawa H (1985) Preoperative imaging of liver metastases. Comparison of angiography, CT scan and ultrasonography. *Ann Surg* **202**: 537–539

Jenkins P and Williams R (1980) Fulminant viral hepatitis. *Clin Gastroenterol* **9**: 171

Martin JK, O'Connell MJ, Wieand HS, Fitzgibbons RJ Jr, Mailliard JA, Rubin J, Nagorney DM, Tschetter LK and Krook JE (1990) Intra-arterial floxuridine vs systemic fluouracil for hepatic metastases from colorectal cancer. *Arch Surg* **125**: 1022–1027

Piedbois P, Buyse M, Kemeny N, Rougier P, Carlson R, Allen-Mersh TG, O'Connell M, Chang A, Sondak V, Kemeny M and Levi E (1996) Reappraisal of hepatic arterial infusion in the treatment of non-resectable liver metastases from colorectal cancer. *J Nat Cancer Inst* **88**: 252–258

Steele G and Ravikumar TS (1989) Resection of hepatic metastases from colorectal cancer. *Ann Surg* **210**: 127–138

Ueda T, Shimada E and Urakawa T (1994) Serum levels of cytokines in patients with colorectal cancer: possible involvement of interleukin-6 and interleukin-8 in haematogenous metastasis. *J Gastroenterol* **29**: 423–429

Wood CB (1980) Prognostic factors in colorectal cancer. In *Recent Advances in Surgery*, Vol. 10, Taylor S (ed.), pp. 259–280. Churchill Livingstone: London

© Cancer Research Campaign 1997